# Structural and Functional Cortical Connectivity Mediating Cross Education of Motor Function

**DOI:** 10.1523/JNEUROSCI.2536-16.2017

**Published:** 2017-03-08

**Authors:** Kathy L. Ruddy, Alexander Leemans, Daniel G. Woolley, Nicole Wenderoth, Richard G. Carson

**Affiliations:** ^1^School of Psychology, Queen's University Belfast, Belfast, BT7 1NN United Kingdom,; ^2^Trinity College Institute of Neuroscience and School of Psychology, Trinity College Dublin, Dublin 2, Ireland,; ^3^Neural Control of Movement Laboratory, ETH, Zurich, 8057 Switzerland,; ^4^Image Sciences Institute, University Medical Center Utrecht, Utrecht, 3584 CX The Netherlands, and; ^5^Movement Control and Neuroplasticity Research Group, Department of Kinesiology, KU Leuven, 3000 Belgium

**Keywords:** interlimb, motor learning, transfer

## Abstract

Cross-education (CE) is the process whereby training with one limb leads to subsequent improvement in performance by the opposite untrained limb. We used multimodal neuroimaging in humans to investigate the mediating neural mechanisms by relating quantitative estimates of functional and structural cortical connectivity to individual levels of interlimb transfer. Resting-state (rs)-fMRI and diffusion weighted imaging (DWI) scans were undertaken before unilateral ballistic wrist flexion training. The rs-fMRI sequence was repeated immediately afterward. The increase in performance of the untrained limb was 83.6% of that observed for the trained limb and significantly greater than that of a control group who undertook no training. Functional connectivity in the resting motor network between right and left supplementary motor areas (SMA) was elevated after training. These changes were not, however, correlated with individual levels of transfer. Analysis of the DWI data using constrained spherical deconvolution-based tractography indicated that fractional anisotropy and apparent fiber density in tracts connecting bilateral SMA were negatively correlated with and predictive of transfer. The findings suggest that interhemispheric interactions between bilateral SMA play an instrumental role in CE and that the structural integrity of the connecting white matter pathways influences the level of transfer.

**SIGNIFICANCE STATEMENT** Strength or skill training with one limb also brings about improvements in the performance of the opposite, untrained limb. This phenomenon, termed cross-education (CE), has obvious potential for the rehabilitation of functional capacity that has been lost through brain insult or musculoskeletal injury. The neural mechanisms that give rise to CE are, however, poorly understood. We used a combination of neuroimaging methods to investigate the pathways in the human brain that mediate CE. We determined that the supplementary motor area (SMA) plays an important role in the interlimb transfer of performance gains and demonstrate that the quality of the white matter fibers connecting right and left SMA predicts the benefit that an individual derives from CE.

## Introduction

Cross-education (CE) is the process whereby training of one limb gives rise to increases in the subsequent performance of its opposite counterpart. Motor learning thus “transfers” from a trained to an untrained effector after a period of practice that is exclusively unilateral. A general conjecture has been that bilateral cortical activity generated during the unilateral training drives concurrent neural adaptations in both cerebral hemispheres (Hellebrandt, 1951). Among the various regions of the cortical motor network that may play an instrumental role in mediating this effect, attention has thus far been focused predominantly upon primary motor cortex (M1).

Although elevated neural activity is evident in ipsilateral M1 during unilateral movements (Kobayashi et al., 2003), larger increases are typically registered in ipsilateral premotor cortex and the supplementary motor area (SMA) (Koeneke et al., 2004) and cingulate motor area (CMA) (Kermadi et al., 2000). In generating hypotheses regarding the neural interactions that mediate CE, anatomical connectivity imposes a necessary constraint. For dorsal premotor cortex (PMd), SMA, and CMA, the densest structural (white matter) connections are with the homologous region in the opposite hemisphere (Ruddy et al., 2016a). Indeed, in view of their structural connectivity profiles, they exhibit a potential to mediate interlimb transfer of performance at least equivalent to that of the primary motor areas.

SMA is involved in movement planning (and sequence encoding; Tanji and Shima, 1994) and plays a prominent role in configuring the neural commands that are necessary to bring about the precise timing of force output (Haller et al., 2009). It also exerts control over both contralateral and ipsilateral limbs (Brinkman and Porter, 1979; Goldberg, 1985; Montgomery et al., 2013). Existing work by Perez et al. (2007a) revealed that, in the context of a motor sequence learning task, fMRI activity in SMA was greatest when the skill had transferred well compared with when it had transferred poorly. Subsequently, they demonstrated that using transcranial magnetic stimulation to perturb SMA during skill acquisition abolished transfer without affecting performance gains for the training limb. Recently, diffusion weighted imaging (DWI) has revealed that white matter streamlines between left and right SMA are more prevalent than those between any other pair of regions in the cortical motor network (Ruddy et al., 2016a).

By virtue of its rich interhemispheric connectivity, which is second only to that of SMA (Ruddy et al., 2016a), PMd gives bilateral effect to the lateralized functions of various regions including prefrontal cortex, parietal cortex, and striatum. Consistent with this role, the microstructural organization of its interhemispheric projections is more coherent and dense than that of any other part of the cortical motor network (Ruddy et al., 2016a). The firing rate of neurons in this region, when recorded directly in primate models, relates to ipsilateral movement parameters such as acceleration and velocity (Kubota and Hamada, 1978).

A high proportion of CMA neurons also exhibit activity that is modulated when the ipsilateral hand is engaged (Kermadi et al., 2000). Because the neural activity registered in CMA appears proportional to the effort that is exerted (Winterer et al., 2002), it might be supposed that this region also plays a role in mediating interlimb transfer, particularly for those tasks that demand maximal motor output. CMA also exhibits dense projections to the homologous region in the opposite hemisphere (Ruddy et al., 2016a).

We used multimodal neuroimaging to investigate the cortical regions that mediate CE of motor function by relating quantitative estimates of functional and structural cortical connectivity to the individual levels of interlimb transfer exhibited in a ballistic movement task. As a first step, resting-state (rs)-fMRI was used to identify elements of the cortical motor network that exhibit changes in functional connectivity as a consequence of unilateral training. Restricting consideration to this subnetwork, we used constrained spherical deconvolution (CSD) based tractography to determine whether individual variations in structural connectivity predicted levels of interlimb transfer.

## Materials and Methods

### 

#### Participants

Twenty-four healthy volunteers (age 22.5 ± 3.20 SD, 16 female) participated in the main experiment, which was composed of an initial session followed by a retention test conducted 7 d later. For these participants, both rs-fMRI and DWI scans were performed in conjunction with the behavioral protocol. An additional group of 21 participants (age 22.0 ± 2.4 SD, 12 female) subsequently participated in a replication experiment and underwent the DWI scanning procedure and behavioral protocol (without tests of retention). Fifteen of these participants were scanned before participating in the behavioral protocol; the others, ∼6 weeks later. A further 20 healthy volunteers (age 22.85 ± 2.06 SD, 9 female) participated in a control experiment that involved only behavioral testing. All participants were right handed according to the Edinburgh Handedness Inventory (Oldfield, 1971) and gave informed consent to procedures approved by the relevant Queen's University Belfast and Trinity College Dublin Ethics Committees, which were conducted in accordance with the Declaration of Helsinki.

#### MRI scanning procedures

The first group of participants in the main experimental group underwent two separate scan sessions on the same day. The first was conducted before behavioral training. Images were acquired on a 3 T Philips Achieva MRI scanner with an eight-channel head coil. A high-resolution T1 anatomical scan was acquired, followed by a DWI scan consisting of a single-shot echoplanar imaging (EPI) sequence with a slice thickness of 2.29 mm, repetition time = 9994 ms, echo time = 73 ms, number of diffusion directions = 61, b value = 1500, number of slices = 60 (transverse), in-plane resolution 2.29 × 2.29 × 2.29 mm^2,^ with a field of view of 258 mm (RL) × 258 mm (AP) × 138 mm (FH).

Thereafter, a 7 min resting-state functional connectivity scan was conducted, consisting of a descending gradient EPI pulse sequence for T2-weighted images (TE = 27 ms, TR = 2000 ms, flip angle = 90°, voxel size 3 × 3 × 3.2 mm), to collect 37 transverse slices, each 3.2 mm thick. Participants were requested to lie still with eyes open, fixate on a cross in front of them, and think about nothing in particular.

Immediately after the cessation of behavioral training, the participants were returned to the scanner, and a second 7 min resting-state scan was performed. To minimize motor activity, they were transported to the scanner bed on an MR-compatible wheelchair. The interval between the end of training and repositioning on the MRI scanner bed was ∼3 min. During this period, the participants closed their eyes and did not engage in conversation.

#### Apparatus and procedures for behavioral task

The left limb executed the training movements. The performance of the right limb was tested before and after training. The participants were seated with forearms supported and stabilized in a neutral position with the elbows semiflexed (100–120°). The angle between the upper arm and the torso was 15–20°. An orthopedic neck brace stabilized the head at ≈15° relative to the sagittal plane during behavioral testing/training.

The hands were secured at midpalm in manipulanda (instrumented to transduce angular displacement) mounted coaxially with the (flexion–extension) axes of rotation of the wrists. A contact switch was activated upon flexion of the wrist (from a neutral position), which was opposed by a stiffness load (≈0.67 Nm/θ − rad).

An opaque screen (depth 50 cm × height 90 cm) was aligned with the participant's sagittal plane. A white cross on the screen served as a point of fixation. This arrangement prevented direct vision of either limb. The EMG activity of flexor carpi radialis (FCR) and extensor carpi radialis longus was recorded from both arms using bipolar surface electrodes. EMG signals were amplified and band-pass filtered (100 Hz to 1 kHz, second-order Butterworth). These and the transducer-derived voltages corresponding to displacement of the wrist were digitized at 2000 Hz.

#### Behavioral paradigm

During the course of training, each participant was required to undertake 300 “fast as possible” discrete flexion movements of the left wrist. Each movement was cued by the presentation of a tone (400 Hz sine wave, 1 s duration). It was made clear to the participants that this was not a reaction time task and that they could initiate the movement in their own time and to whatever magnitude within the 0–90° flexion range that was comfortable for them. In the course of a “trial,” 10 such movements were performed. Successive movements were separated by ≈7000 ms intervals. There were 15 trials in each of two “blocks” (total 300). Within each block, successive trials were separated by 30 s intervals. Five practice movements were first undertaken to familiarize the participant with the procedure.

Throughout training, the participants were encouraged to increase the peak acceleration of their wrist flexion movements continually. Feedback of performance was provided immediately after each movement (with the exception of the first two) by means of two qualitatively distinct auditory “sound bites.” One indicated that the peak acceleration of the movement was greater than the mean of the two preceding attempts. The other indicated that the mean peak acceleration of the preceding two attempts had not been surpassed. Participants were instructed as follows: “Each movement should be your fastest possible. Listen to the auditory feedback to determine whether you are improving or not. The aim is to hear the 'positive' sound bite as often as possible, which indicates that your maximum is improving.”

Before the commencement of left limb training, each participant performed 10 “fast as possible” discrete flexion movements of the right wrist. No feedback of performance was provided after these movements. A further series of 10 right limb movements were performed 10 min after completion of the first block of (150) training movements undertaken by the left limb and 10 min after completion of the second block of (150) training movements undertaken by the left limb. In tests of retention conducted 7 d later, the participants performed a further 10 movements of the right wrist, followed by 10 movements of the left wrist. No feedback of performance was provided during these tests.

#### Control experiment

Training of the left limb was not undertaken. The performance of the right limb was, however, assessed in the manner described previously. In the period during which left limb training would otherwise have occurred, participants listened to the auditory tones and soundbites used in the full training protocol. In this case, the soundbites were presented in a random order. To maintain an equivalent level of attention, the participants were required to count and subsequently report the number of times that a particular soundbite occurred during each trial. This group did not undergo any scanning procedures.

#### Data processing and analysis

##### Kinematic data.

After digital filtering (second order, dual-pass Butterworth, low-pass 6 Hz), the transducer-derived displacement signals were differentiated twice to derive acceleration. The peak acceleration in wrist flexion was obtained for each movement. The mean peak acceleration of the 10 movements performed in each trial was then calculated.

The increases in performance (i.e., in peak acceleration) exhibited by the participants through training were not always monotonic. The change in performance of the training (left) limb was therefore calculated as follows: the mean peak acceleration of the trial (containing 10 movements) wherein they performed best minus the mean peak acceleration of the first 10 movements (i.e., trial 1), expressed as a percentage of the mean peak acceleration of trial 1. The change in performance of the untrained (right) limb was calculated as the mean peak acceleration of the 10 movements executed after the completion of left limb training (i.e., the post-trial) minus the mean peak acceleration of the 10 movements executed before the commencement of left limb training (i.e., the pre-trial), expressed as a percentage of the mean peak acceleration of the pre-trial.

To eliminate participants who did not engage fully with the training task in the fashion intended, we excluded from further analysis those who failed to exhibit improvements in performance (>15% left limb; >0% right limb) above levels defined in a previous study in which a very large sample (*n* = 117) was used (Ruddy et al., 2016b). The magnitude of interlimb transfer was calculated for the remaining participants as the change in performance of the untrained (right) limb, expressed as a percentage of the change in performance of the training limb.

##### Functional localizer.

**Figure 1.**
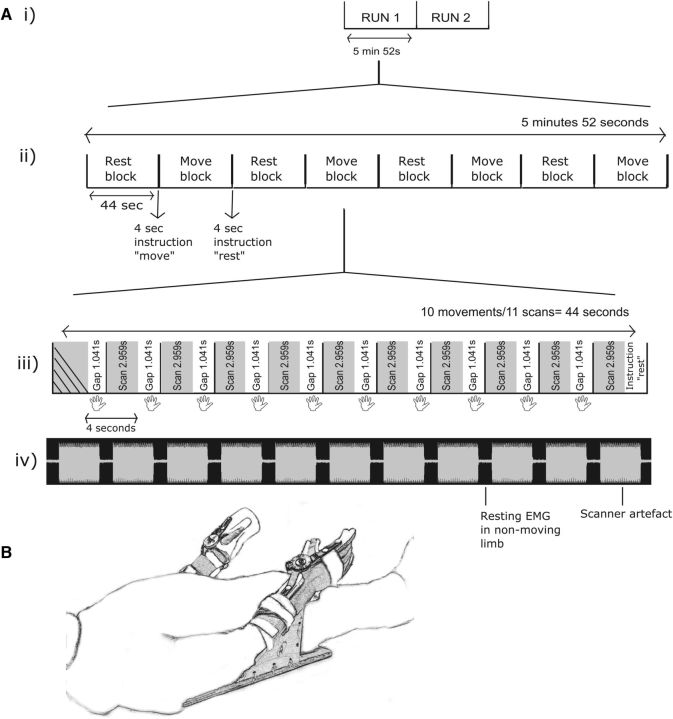
Functional localizer design. ***Ai***, Two identical fMRI runs executed for each participant with a duration of 5 min 52 s per scan. ***Aii***, Block design for one scanning run containing four movement and four rest blocks interspaced by recorded (timed) verbal instructions. ***Aiii***, Time course of one fMRI movement block using the sparse sampling sequence in whith the TR of 4 s is composed of a 2.959 s scan and 1.041 s silent gap. Movements (indicated by the hand icon) were executed in the gap period (10 movements). ***Aiv***, One participant's EMG recording for one movement block. The scanner artifact can be clearly seen during scanning, followed by the silent gap during which EMG was recorded from the nonmoving limb. ***B***, MRI-compatible apparatus that was used for the task.

To aid with the definition of ROIs for the subsequent connectivity analyses, a functional localizer scan was performed in a separate session on a subset of 10 participants who had previously completed the behavioral protocol (aged 23 ± 2.7 SD, 8 female). Participants lay supine in the scanner attending to a fixation cross on screen, with arms supported on a custom-made wooden apparatus that provided stabilization of the upper and lower arm segments (Fig. 1). The hand and forearm were encased in a plastic, jointed orthosis such that the only mechanical degree of freedom was flexion–extension of the wrist. An elastic system that applied force in opposition to wrist flexion was incorporated into the device to simulate the conditions of the experimental protocol undertaken outside the scanner. EMG recordings were taken from the right (nonmoving) FCR during scanning using a specialized MR-compatible EMG recording system (Biopac Systems) and MR-compatible surface electrodes. EMG signals were amplified, band-pass (100 Hz to 1000 Hz) filtered, and digitized at 2000 Hz. These signals were later inspected to verify that the right FCR remained quiescent during movements of the left limb.

T2 weighted functional images were acquired using the following EPI sparse sampling pulse sequence: 59 transverse slices, slice thickness = 3 mm, slice gap = 0.05 mm, TE = 32 ms, TR = 4000 ms, flip angle = 90°, matrix = 80 × 80, voxel size 3 × 3 × 3 mm. Within the 4000 ms TR sparse sampling sequence, the actual scan time was 2959 ms, with a “silent gap” of 1041 ms. The protocol consisted of two scanning runs, each containing four movement blocks and four rest blocks. Movement and rest blocks were alternated, with each run beginning with a rest block. Within each movement block, the participant was required to execute 10 fast as possible wrist flexion movements with the left limb in response to a sequence of tones (presented at 4000 ms intervals). Each tone was presented at the beginning of the silent gap so the resultant movement was completed before the beginning of the following scan. The advantage of using the sparse sampling sequence in this way is twofold. First, the presentation of the auditory tone in the silent gap allows it to be heard clearly by the participant rather than being obscured by the noise of the scanner. Second, EMG recordings taken during scanning are subject to artifacts arising from magnetic interference, rendering the low-amplitude signals detected from the muscles difficult to discern. Because all movements were executed in the silent gap, the EMG recordings obtained during this interval were of a quality sufficient for analysis. Because the hemodynamic lag in the BOLD signal can be up to 5–7 s after neural activity, the execution of movements during the silent gap does not compromise the signal quality. During rest blocks, the same auditory stimuli were presented. Prerecorded verbal instructions to “move” or “rest” indicated the beginning of movement blocks and rest blocks. In total, there were 40 movements in each run and 40 equivalent rest periods. All sessions in the scanner were video recorded and checked subsequently to verify that movements were made in the designated periods.

##### Functional localizer analysis.

Preprocessing and analysis of functional scan data were implemented using SPM8 (Wellcome Department of Imaging Neuroscience, University College, London). EPIs were spatially realigned to the mean EPI image, slice time corrected to account for differences in slice acquisition time by temporal interpolation to the middle slice (reference slice = 30), and spatially coregistered to the individual's high-resolution anatomical T1 image. Anatomical images were segmented and the resulting transformation parameters were used to normalize the realigned EPI images to the Montreal Neurological Institute (MNI) template. Normalized functional images were then smoothed using an isotropic 8 mm full-width at half-maximum Gaussian kernel.

Individual participant data were entered into a first-level analysis performed on a voxel-by-voxel basis in the context of a general linear model. “Movement” and “rest” were the two alternating components of the block design, which was convolved with the canonical hemodynamic response function. At the second level, a fixed-effects analysis was performed including all runs across all participants (20 runs total).

##### Creation of ROIs.

The activation mask resulting from the fixed-effects model was thresholded at a T value corresponding to *p* = 0.05, FWE corrected. This mask was then used to identify areas of overlap with ROIs delineated a priori from a selection of probabilistic brain atlases, each thresholded at 50%. The locations of SMA and cingulate cortex were taken from the Harvard Oxford cortical atlas. The Jülich histological atlas (Eickhoff et al., 2005, 2006, 2007) was used to differentiate between primary motor (anterior and posterior, M1a and M1p) and premotor regions because the border between these distinct areas may only be differentiated reliably by cytoarchitectural means. Because the Jülich “premotor” area also encompasses regions of cortex otherwise known as SMA, the Harvard Oxford SMA region was subtracted from the Jülich premotor region to ensure that there was no overlap between these two ROIs. Using these atlas regions, only voxels falling within the overlap area with the thresholded activation mask were considered part of the ROI. Because the current investigation was focused exclusively on cortical mechanisms, activation clusters in the cerebellum and subcortical regions were not considered as ROIs.

As the participants performed a unilateral (left limb) movement during the functional localizer, it was anticipated that there would be minimal or no detectable activation in some ipsilateral (left hemisphere) motor regions. Guided by an appreciation that the cerebral hemispheres should not be considered symmetrical and that the extent of the asymmetry is greater for “nonprimary” than for “primary” regions (Zuo et al., 2010), we considered that it was inappropriate to simply mirror the ROIs that were detected in the right hemisphere onto homologs in the left hemisphere. Therefore, to find corresponding ROIs in the left hemisphere to match those in the right, we conducted a “seed-to-voxel” analysis based on the resting-state connectivity data recorded before training (i.e., at baseline). Seeds for this connectivity analysis were the right hemisphere ROIs already derived from the functional localizer. Connectivity was assessed at an FWE-corrected α of 0.01 within a restricted search region in the left hemisphere that corresponded to the appropriate atlas mask (e.g., Harvard Oxford or Jülich). For each left hemisphere ROI, this process was performed from all of the five right hemisphere ROIs (M1a, M1p, SMA proper, CMA, and PMd) and the five resulting masks were combined to derive the cortical region that displayed interhemispheric motor connectivity with all of the contralateral regions. In the interests of consistency, all five of the left hemisphere ROIs were created using this approach and all right hemisphere ROIs were derived from the functional localizer activation. Average ROI volume was 3623 mm^3^ (range 297–15741).

##### Resting-state data analysis.

The incidence of head movements was assessed using custom-written MATLAB software based on the method described by Power et al. (2012). We calculated framewise displacement, which is defined as the sum of the absolute scan to scan difference of the six translational and rotational realignment parameters. If >25% of all rest scans for an individual participant exceeded a framewise displacement of 0.5 mm, then the participant was excluded from further analysis. Preprocessing was implemented using SPM8. EPIs were spatially realigned to the mean EPI image, slice time corrected to account for differences in slice acquisition time by temporal interpolation to the middle slice (reference slice = 19), and spatially coregistered to the individual's high-resolution anatomical T1 image. Anatomical images were segmented and the resulting transformation parameters were used to normalize the realigned EPI images to the MNI template. Normalized functional images were then smoothed using an isotropic 6 mm full-width at half maximum Gaussian kernel.

After preprocessing, analysis was performed using the Functional Connectivity Toolbox version 14. Changes in resting functional connectivity occurring between pre-training and post-training were assessed in the context of a semipartial correlation analysis. Resting-state images were first band-pass filtered (0.008 Hz < f < 0.09 Hz, rectangular FFT temporal filter equivalent to Butterworth *n* = inf). In addition to regressing out the 3D motion parameters, we also included regressors to deweight scans with a framewise displacement >0.5 mm. White matter, CSF, and the time course of the posterior cingulate cortex were also removed as confounds after the CompCor strategy (Behzadi et al., 2007).

##### ROI–ROI analysis.

Hypotheses regarding the potential functional interactions that mediate transfer of performance between the limbs were informed by prior knowledge. With respect to the five bilateral regions that were the focus of attention (SMA, CMA, PMd, M1a, and M1p), it has been surmised that the scope for direct interhemispheric interactions decreases progressively along a functional gradient that culminates with those that have the most prominent role in generating motor output (Carson, 2005). Accordingly, for SMA, PMd, and CMA, the densest structural connections are with their homologs in the opposite hemisphere and then generally with the other members of this set. Projections from SMA, PMd, and CMA to primary motor regions (M1a and M1p) in the opposite hemisphere are scarce (Ruddy et al., 2016a). Therefore, we investigated 11 pairs of ROIs comprising all possible homologous pairs (*n* = 5) plus bilateral nonhomologous pairs composed from SMA, PMd, and CMA (*n* = 6). Changes in functional connectivity from pre-training to post-training were evaluated for these 11 pairs of ROIs (Table 1). Only ROI pairs associated with large effect sizes (Cohen's *d* >0.8) were selected for subsequent analysis of structural connectivity.

**Table 1.** Pre-training to post-training change in resting functional connectivity

Planned contrast	Beta	T(17)	Effect size (Cohen's *d*)
RM1a−LM1a	0.01	0.3	0.15
RM1p−LM1p	0	−0.07	0.03
RPMd−LPMd	0.01	0.32	0.16
RSMA−LSMA	0.05	1.72	0.83
RCMA−LCMA	0.01	0.32	0.16
RCMA−LPMd	0.03	1.03	0.50
RCMA−LSMA	−0.01	−0.23	0.11
RPMd−LCMA	0.02	0.54	0.26
RPMd−LSMA	−0.04	−1.17	0.57
RSMA−LPMd	−0.03	−1.24	0.60
RSMA−LCMA	0.01	0.16	0.08

Should are planned contrasts between nodes in the right and left hemisphere based upon a priori defined anatomical hypotheses.

##### DWI analysis.

DWI data were processed using ExploreDTI (Leemans et al., 2009). Images were corrected for subject motion and eddy currents using the procedure described in Leemans and Jones (2009). Tensor estimation was then performed using the iteratively reweighted linear least-squares approach (Veraart et al., 2013). Whole-brain tractography was performed using CSD incorporating a recently developed approach to optimize calibration of the DW signal response function, which has been shown to provide a more accurate estimation of the fiber orientation distribution in each voxel than previous methods of response function estimation (Tax et al., 2014).

A restricted tractography analysis was performed subsequently to reconstruct streamlines passing through bilateral ROIs that were discerned from the resting-state functional connectivity analysis. For each of the reconstructed streamlines, the median apparent fiber density (AFD) and fractional anisotropy (FA) were taken as outcome measures. AFD is a highly sensitive measure of microstructural organization that is thought to reflect the density of the underlying pathways reliably, even in regions with complex fiber architecture (Dell'Acqua et al., 2013; Raffelt et al., 2012). Because the biological relevance of FA values is becoming increasingly questioned, we also report this measure simply to facilitate comparison with other studies.

#### Relationships between functional and structural connectivity indices and transfer

To establish whether there were relationships between the degree of CE (i.e., interlimb transfer of performance) exhibited by individual participants and indices of functional and structural connectivity, statistical measures of association (correlation and regression) were derived. Because it is well documented that least-squares variants of these measures are extremely sensitive to the presence of univariate and bivariate outliers, robust methods (Wilcox, 2012) were used in all cases. Confidence intervals (95%) derived using a bootstrapping approach are reported for the measures of association.

## Results

### Removal of participants due to head movements

On the basis of the aforementioned head movement screening criteria for resting-state data, two participants were removed from the neuroimaging and behavioral analyses.

### Performance of the untrained limb

**Figure 2.**
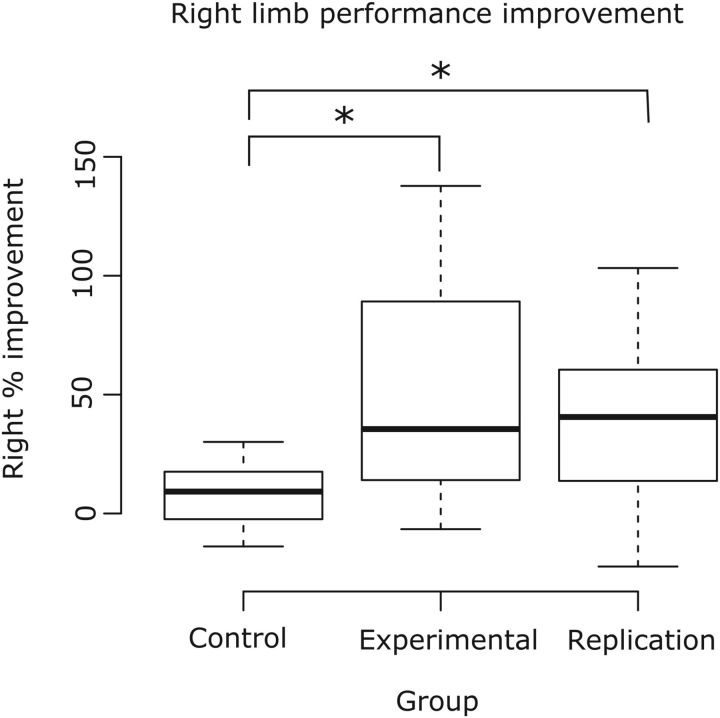
Boxplots representing right limb performance. Three different groups of participants are represented; the control group who did not undergo left limb training (*n* = 20), the main experimental group (*n* = 22), and the separate replication group (*n* = 21). There is a significant difference between each of the two groups who performed training and the control group who did not. Statistics and boxplots conducted before the removal are behavioral outliers.

For the 20 participants in the control group who did not engage in left limb training, the mean peak acceleration of movements generated by the right limb increased marginally (median = 9.21%, IQR = −2.23 to 16.42). This change was markedly smaller than that exhibited by those in the main experimental group (median = 35.50%, IQR = 14.04–89.17, Mann–Whitney *U* = 79, df = 40, *p* < 0.001, *r* = 0.53) and the replication group (median = 40.58%, IQR = 13.72–60.50, Mann–Whitney *U* = 86, df = 39, *p* < 0.001, *r* = 0.51) (Fig. 2). On the basis of these findings, it can be concluded that the improvement in right limb performance exhibited by the participants in the main experimental group and replication group was attributable to the training movements performed by the opposite limb, rather than being invoked by repetition of the assessment procedure. Before calculating the degree of CE (i.e., interlimb transfer of performance), four outliers were removed from the main experimental group (remaining *n* = 18) and four from the replication group of participants (remaining *n* = 17) on the basis of the behavioral data screening methods described previously.

### Transfer to the untrained limb

**Figure 3.**
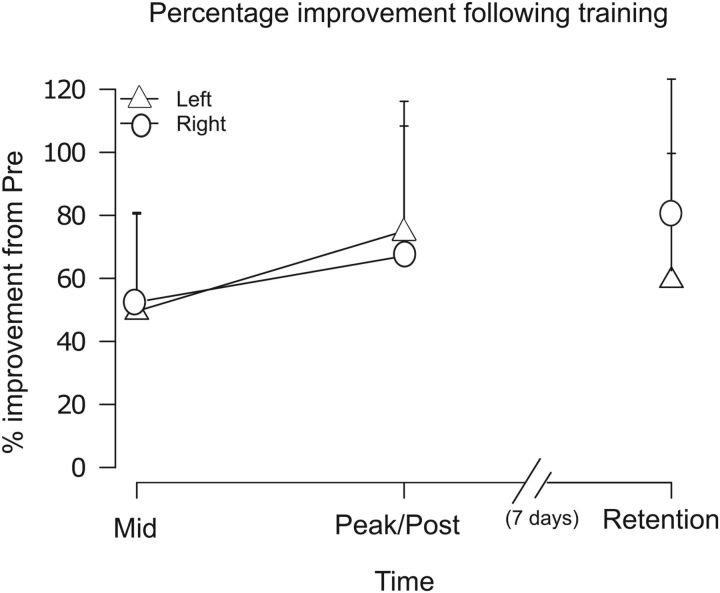
Performance improvement of right and left limbs. The performance of the right limb (circular symbols) was assessed at the midpoint of (left limb) training and post-training. Improvements in performance registered at these times were defined as the change in acceleration relative to the starting (pre-training) value expressed as a percentage of the starting value. Necessarily, factors such as fatigue will have exerted an accumulating effect on the performance of the training limb (triangular symbols). Therefore, rather than examining the final training trial, the “peak” improvement in performance was taken to be the difference between the value derived from the trial in which the highest mean acceleration was obtained and that of the first training trial, expressed as a percentage of the mean acceleration for the first training trial. Transfer was calculated as the change in performance of the untrained (right) limb expressed as a percentage of the peak change in performance of the training limb. The corresponding transfer values are reported in the text. In a retention test undertaken 7 d later, the performance of both the trained (left) and untrained (right) limb was again assessed.

The level of transfer (i.e., change in performance of the untrained limb expressed as a percentage of the change in performance of the training limb) exhibited by the participants in the main experimental group was 83.63% (median, IQR = 59.96–132.68). In a retention test conducted 1 week later, the median level of transfer was 109.67% (IQR = 77.53–145.33) (see Fig. 3 for performance improvement of left and right limbs). For the participants in the replication group, the median immediate level of transfer was 95.34% (IQR 66.48–121.02).

### Functional localizer results

**Figure 4.**
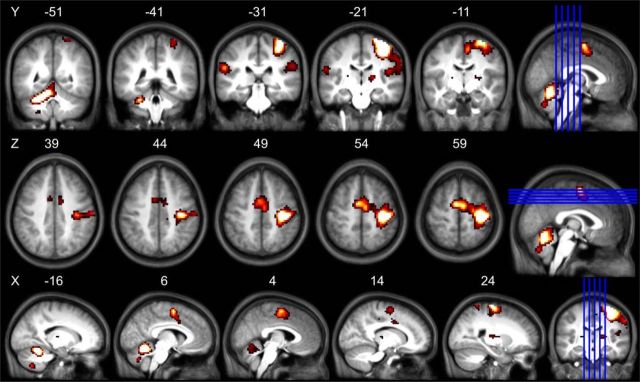
fMRI localizer activation maps. Slices are shown with MNI coordinates. Results are FWE corrected at an α value of *p* = 0.05. Multiple slice views in coronal, axial, and sagittal planes are displayed on an averaged T1 from all 18 resting-state participants. Blue lines indicate location of slices shown.

**Figure 5.**
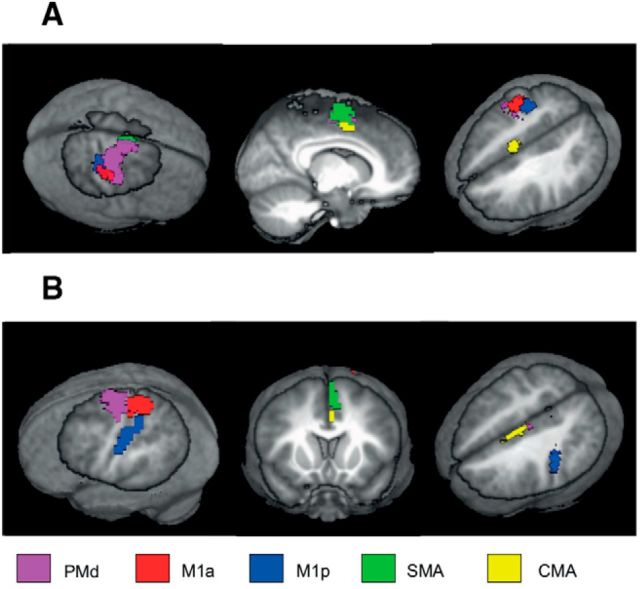
ROIs used for functional connectivity analysis. ***A***, Right hemisphere ROIs derived from the functional localizer fMRI data. ***B***, Left hemisphere ROIs derived from using a seed-to-voxel analysis on the baseline resting-state data to derive corresponding ROIs that exhibited functional connectivity with each of the contralateral motor regions. ROIs are displayed on a rendered view of an averaged T1 from all 18 participants.

The T value above which activated voxels were considered significant was T = 4.7, which corresponded to *p* = 0.05, FWE corrected. The activation mask was thresholded at this level and overlapped with each of the aforementioned atlas masks, resulting in five right hemisphere ROIs corresponding to SMA proper, PMd, CMA, M1a, and M1p. Activation was also present in left (ipsilateral to movement) SMA(proper, CMA, and M1 and in left cerebellum and bilateral thalamus (Fig. 4). See Figure 5 for final ROIs based upon this functional data and for those derived from the previously described seed-to-voxel analysis for creating left hemisphere ROIs.

### Change in functional connectivity pre-training to post-training

**Figure 6.**
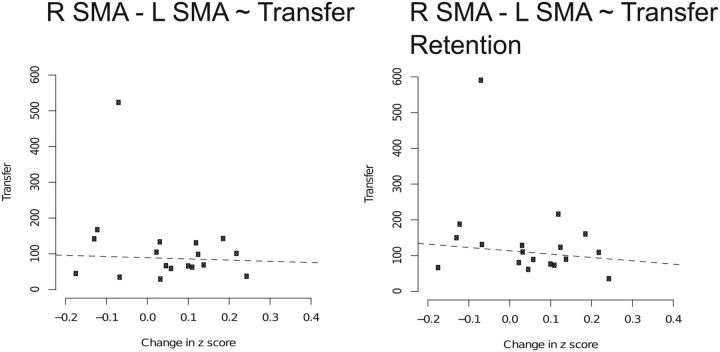
Scatterplots indicating lack of correlation between change in functional connectivity and transfer. Line of best fit is derived from the corresponding robust regression model. Change in connectivity is quantified by a change in *Z* scores pre-training to post-training. Transfer was measured at post-training and also 1 week later at a retention test. Dashed lines indicate nonsignificant associations.

Functional connectivity between right SMA proper and left SMA proper increased reliably from pre-training to post-training (β = 0.05, T(17) = 1.72, *d* = 0.81; Table 1). The corresponding changes in *Z*-score obtained for the individual participants were not, however, correlated with or predictive of transfer of performance when this was assessed either acutely or in the retention test undertaken 1 week later (associated *r*_pb_ values, −0.08 to −0.18, *p* = 0.28–0.79; Fig. 6). There were no other instances in which functional connectivity between the specified nodes of the cortical motor network was observed to increase (Table 1).

### Structural connectivity association with transfer

To optimize the inferential power of our analysis of the tractography data, we made the a priori decision to restrict consideration to those connections between nodes in the cortical motor network for which training-related increases in functional connectivity were obtained. In practice, this meant that only tracts connecting left SMA to right SMA were examined.

**Figure 7.**
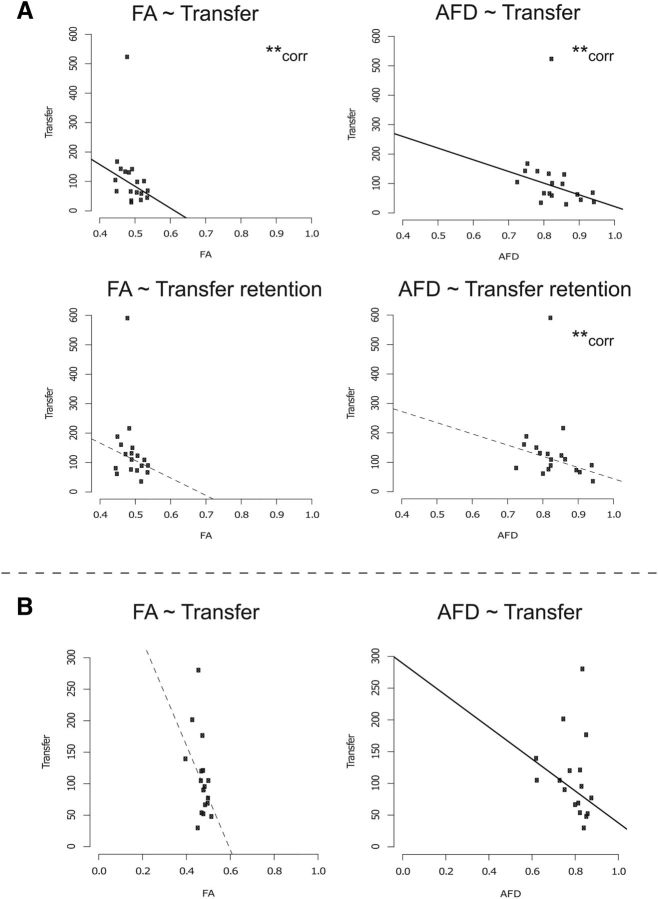
Scatterplots representing DWI measures for tracts connecting bilateral SMA and their associations with transfer. ***A***, Significant associations with transfer for the main experimental group (*n* = 18). Both FA and AFD are negatively correlated with and predictive of transfer. AFD was still predictive of transfer at retention. ***B***, Associations between the DWI measures and transfer for the replication group of 17 participants. Similar to the first group, AFD is a significant predictor of transfer. Note that the outliers do not impact upon the line of best fit resulting from the robust regression analysis. Solid lines represent that the regression result was significant. Dashed lines indicate nonsignificant associations. **corr denotes cases when the correlation is significant even if the regression is not.

There was a striking association between the measures of structural connectivity obtained for this fiber bundle and the degree of CE exhibited by individual participants. The apparent fiber density AFD was negatively correlated with (*r_pb_* = −0.57, TS = −2.75, *p* = 0.01, *n* = 18, CI = −0.80–0.10) and predictive of (regression: slope = −396.80, *t* = −3.55, df = 16, *p* = 0.002, CI = −678.52–113.37) transfer acutely, and remained correlated with (but not predictive of) the transfer evident in the retention test conducted 1 week later (*r_pb_* = −0.48, TS = −2.19, *p* = 0.04, *n* = 18, CI = −0.81 to 0.10; regression: slope = −380.91, *t* = −1.81, df = 16, *p* = 0.09, CI = −684.82 to 138.28; Fig. 7*A*). FA was negatively correlated with and predictive of transfer of performance acutely (*r_pb_* = −0.51, TS = −2.35, *p* = 0.03, *n* = 18, CI = −0.79 to −0.10; regression: slope = −741.35, *t* = −2.50, df = 16, *p* = 0.02, CI = −1409.72 to −28.45), but not with transfer measured in the retention test conducted 1 week later (associated *p*-values = 0.12–0.23). The number of reconstructed streamlines was not correlated with or predictive of transfer either acutely or when assessed in the retention test (associated *p*-values = 0.26–0.91).

### Replication analysis of structural connectivity

The principal features of these associations were also present in the additional group of 17 participants comprising the replication experiment. Once again, higher AFD values for fiber tracts connecting left SMA to and right SMA were predictive of lower levels of transfer (regression: slope = −251.38, *t* = −2.72, df = 15, *p* = 0.02, CI = −560.31 to 146.25; Fig. 7*B*). The negative association between FA in tracts connecting bilateral SMA and the level of transfer closely approached statistical significance (*r_pb_* = −0.46, TS = −2.00, *p* = 0.06, *n* = 17, CI = −0.85 to 0.14; regression: slope = −829.11, *t* = −2.08, df = 15, *p* = 0.05, CI = −2379.73 to 983.13). Again, the number of reconstructed streamlines was neither correlated with nor predictive of transfer (associated *p*-values = 0.41–0.49).

## Discussion

On the grounds of their structural and functional connectivity profiles, we surmised that SMA, CMA, and PMd each exhibit a potential to mediate interlimb transfer of the performance gains realized through unilateral practice that is at least equivalent to that of the primary motor regions (i.e., M1a and M1p). In the context of a ballistic movement training task used to examine this possibility, we observed that the performance (peak acceleration) of the untrained (right) limb increased by >35%. This corresponded to >83% of the gains in performance realized by the training limb. In conjunction, rs-fMRI connectivity between right and left SMA was seen to increase. This was not the case for projections between other nodes of the cortical motor network. Although changes in SMA–SMA functional connectivity did not correlate with or predict individual levels of interlimb transfer, the net alteration in the state of this pathway signified its possible role in mediating the adaptive response to the training regime. Restricting our attention to structural estimates of fiber bundles connecting left and right SMA (i.e., DWI-based CSD analysis) therefore, we determined that FA and AFD were strongly and negatively associated with and predictive of transfer. In other words, individuals with higher FA and AFD for SMA–SMA streamlines exhibited lower levels of interlimb transfer. The expression of this specific structure–function association was examined in a further independent group of participants, for whom the same negative relationship was obtained.

In a previous investigation by Fling et al. (2011), a similar inverse relationship was reported in the context of bimanual movement control in young adults. In the midbody of the corpus callosum, greater microstructural integrity of the region containing interhemispheric sensorimotor fibers was associated with poorer performance on a complex bimanual task. It is intriguing that this finding likewise demonstrates a counterintuitive relationship, albeit in a region of the corpus callosum that is different from that in which the SMA–SMA fibers cross. The differences are likely to be accountable for in terms of the task that was studied and the measures of performance that were the focus of interest. In the present study, the association was present for interlimb transfer of performance, rather than for level of performance per se.

It has long been assumed that SMA (along with PMd and ipsilateral M1) is the key component of a “nonmirroring network” that prevents unwanted movement in the limb contralateral to that which is the focus of (intentionally unilateral) action (Beaulé et al., 2012). Although, in general, DWI does not permit determination of whether the neurons indirectly portrayed by the technique are facilitatory or inhibitory, callosal neurons are a special case. They are glutamatergic (Werhahn et al., 1999) and facilitatory to their immediate targets (Houzel and Milleret, 1999). Therefore, presumably, the individuals in this study who exhibited a low degree of SMA–SMA structural connectivity (the assumption being that low FA and AFD values reflect less densely packed fibers) possessed lesser scope to facilitate immediate target neurons in the opposite SMA (and vice versa). How is this suggestion to be reconciled with the observation that they exhibited greater interlimb transfer than individuals who manifested higher SMA–SMA structural connectivity?

One possibility is that individuals with higher SMA–SMA structural connectivity have a more effective nonmirroring network that suppresses motor overflow during unilateral training and thus reduces the ensuing level of CE. However, the key problem with this explanation is that electrophysiological studies have repeatedly failed to demonstrate a link between motor overflow and interlimb transfer (Carroll et al., 2006; Ruddy et al., 2016b). Therefore, additional mediating factors must be identified to account for the current findings.

The only electrophysiological measure for which reliable association with CE has been demonstrated consistently is the so-called (short-latency) interhemispheric inhibition (IHI). In a variety of task contexts, greater reductions in IHI after training accompany higher levels of interlimb transfer (Perez et al., 2007b; Camus et al., 2009; Hortobagyi et al., 2011). IHI is obtained when an initial magnetic (conditioning) stimulus is applied to one primary motor cortex shortly (6–15 ms) before a second (test) stimulus is directed to the other. In these circumstances, the magnitude of the response to the test stimulus is typically reduced (Ferbert et al., 1992). Because all corpus callosum neurons have a facilitatory action on their immediate targets (in the opposite hemisphere), IHI is most likely mediated through local inhibitory interneurons (Berlucchi et al., 1990). It is not believed that these interneurons project directly onto pyramidal tract neurons within M1 (Daskalakis et al., 2002; Uehara et al., 2013). Therefore, the local neural circuits that regulate the expression of IHI are at least partially distinct from those with the most immediate effect on the postsynaptic state of descending corticospinal projections from M1.

SMA has been described previously as a gateway for interhemispheric motor control, acting as a mediator by regulating the degree of interaction between lateralized elements of the motor network that are directly engaged in generating descending commands (Grefkes et al., 2008). It exhibits dense connectivity with cortical and subcortical motor structures and, by virtue of ipsilateral and contralateral projections, it has the potential to both influence control of the contralateral limb through fibers reaching ipsilateral M1, and modulate the influence of the opposite SMA (and thus M1) through callosal connections (Goldberg, 1985). Within each hemisphere, by far the densest connectivity in the motor network is between SMA and M1 (Goldberg, 1985). It appears plausible, therefore, that the (negative) relationship between SMA–SMA structural connectivity and interlimb transfer revealed in the present study and the previously reported association between the IHI measure and transfer have a common origin. The missing inferential element requires determination of the manner in which SMA–SMA structural connectivity influences variations in the expression of such electrophysiological indices as IHI.

There is, however, a more general challenge: that of relating measures of interhemispheric interactions obtained from conscious humans using noninvasive brain stimulation to those inferred from neuroimaging or derived from animal preparations. It is not clear, for example, that the electrophysiological techniques presently available (e.g., IHI) provide an adequate representation of variations in the local balance between excitation and inhibition that occur in the context of voluntary movements (Ruddy and Carson, 2013). It may at first glance seem counterintuitive, for example, that there is a negative association between SMA–SMA structural connectivity and interlimb transfer and that greater reductions in IHI after training accompany higher levels of interlimb transfer (Perez et al., 2007b; Camus et al., 2009; Hortobagyi et al., 2011). The issue, however, is one of resolving the common mechanistic basis of these phenomena, with the particular objective of establishing the manner in which excitatory and inhibitory interhemispheric interactions and the properties of the anatomical pathways that provide their medium determine the bilateral benefit that an individual derives from unilateral training.

### Limitations of the study and future recommendations

Defining groups of voxels to serve as ROIs was a particular challenge for this investigation because performing the entire interlimb transfer task in the scanner was not feasible and very little existing knowledge is available regarding brain regions involved in transfer of motor learning. Therefore, we adopted the approach of performing the left limb motor task during an fMRI localizer to derive right hemisphere regions involved in the task and subsequently used these “seeds” to reveal the territories in the left (untrained) hemisphere that exhibited resting (task-free) interhemispheric connectivity with these regions. This approach is not without caveats because the resulting ROIs may not be perfectly symmetrical in the two hemispheres. In addition, we performed the localizer task with a subset of participants (*n* = 10) and used a fixed effects model to maximize the power available to extract task-based activation clusters in this small sample. The facility to engage the entire group of participants in the localizer task would have permitted the use of random-effects analyses. Power to detect the motor network during the localizer task might also have been further optimized by jittering the onset of the wrist movements relative to the EPI scan onsets to provide better sampling of the BOLD response in regions with very fast or slow hemodynamic responses (Balsters et al., 2013; O'Connell et al., 2012).

### Concluding remarks

Our findings reveal that SMA–SMA transcallosal structural connectivity, and the extent to which it varies across individuals, is a remarkably good predictor of the interlimb transfer of performance that occurs after unilateral training in the context of this ballistic movement task. Nonetheless, it should not be inferred that this one pathway represents the exclusive basis of CE. We adopted the investigative strategy of testing a restricted set of hypotheses relating to structural connectivity between specific nodes in the cortical motor network. These hypotheses were generated first on an a priori basis informed by our own previous findings and those of others and then refined further on an empirical basis by analyzing task-related changes in functional connectivity. This study was thus not a general exploration of all potential sources of variation in structural brain connectivity that may contribute to individual differences in the magnitude of interlimb transfer. For example, subcortical and intrahemispheric structural connectivity was not considered. Furthermore, whereas our findings suggest that the structural integrity of the white matter pathways projecting between left and right SMA influences the level of interlimb transfer in this task, it remains to be established whether these generalize to other forms of movement. However, it is known that the overt characteristics of the CE phenomenon exhibit a degree of task specificity (Ruddy and Carson, 2013).

## References

[B1] Balsters JH, Robertson IH, Calhoun VD (2013) BOLD frequency power indexes working memory performance. Front Hum Neurosci 7:207. 10.3389/fnhum.2013.00207 23720623 PMC3655325

[B2] Beaulé V, Tremblay S, Théoret H (2012) Interhemispheric control of unilateral movement. Neural Plast 2012:627816. 10.1155/2012/627816 23304559 PMC3523159

[B3] Behzadi Y, Restom K, Liau J, Liu TT (2007) A component based noise correction method (CompCor) for BOLD and perfusion based fMRI. Neuroimage 37:90–101. 10.1016/j.neuroimage.2007.04.042 17560126 PMC2214855

[B4] Berlucchi G, Boller F, Grafman J (1990) Commisurotomy studies in animals In: Handbook of Neuropsychology, Vol. 4 (Boller F, Grafman J, eds), pp. 9–47. Amsterdam: Elsevier.

[B5] Brinkman C, Porter R (1979) Supplementary motor area in the monkey: activity of neurons during performance of a learned motor task. J Neurophysiol 42:681–709. 107282 10.1152/jn.1979.42.3.681

[B6] Camus M, Ragert P, Vandermeeren Y, Cohen LG (2009) Mechanisms controlling motor output to a transfer hand after learning a sequential pinch force skill with the opposite hand. Clin Neurophysiol 120:1859–1865. 10.1016/j.clinph.2009.08.013 19766535 PMC2767461

[B7] Carroll TJ, Herbert RD, Munn J, Lee M, Gandevia SC (2006) Contralateral effects of unilateral strength training: evidence and possible mechanisms. J Appl Physiol 101:1514–1522. 10.1152/japplphysiol.00531.2006 17043329

[B8] Carson RG (2005) Neural pathways mediating bilateral interactions between the upper limbs. Brain Res Brain Res Rev 49:641–662. 10.1016/j.brainresrev.2005.03.005 15904971

[B9] Daskalakis ZJ, Christensen BK, Fitzgerald PB, Roshan L, Chen R (2002) The mechanisms of interhemispheric inhibition in the human motor cortex. J Physiol 543:317–326. 10.1113/jphysiol.2002.017673 12181302 PMC2290496

[B10] Dell'Acqua F, Simmons A, Williams SC, Catani M (2013) Can spherical deconvolution provide more information than fiber orientations? Hindrance modulated orientational anisotropy, a true-tract specific index to characterize white matter diffusion. Hum Brain Mapp 34:2464–2483. 10.1002/hbm.22080 22488973 PMC6870506

[B11] Eickhoff SB, Stephan KE, Mohlberg H, Grefkes C, Fink GR, Amunts K, Zilles K (2005) A new SPM toolbox for combining probabilistic cytoarchitectonic maps and functional imaging data. Neuroimage 25:1325–1335. 10.1016/j.neuroimage.2004.12.034 15850749

[B12] Eickhoff SB, Heim S, Zilles K, Amunts K (2006) Testing anatomically specified hypotheses in functional imaging using cytoarchitectonic maps. Neuroimage 32:570–582. 10.1016/j.neuroimage.2006.04.204 16781166

[B13] Eickhoff SB, Paus T, Caspers S, Grosbras MH, Evans AC, Zilles K, Amunts K (2007) Assignment of functional activations to probabilistic cytoarchitectonic areas revisited. Neuroimage 36:511–521. 10.1016/j.neuroimage.2007.03.060 17499520

[B14] Ferbert A, Priori A, Rothwell JC, Day BL, Colebatch JG, Marsden CD (1992) Interhemispheric inhibition of the human motor cortex. J Physiol 453:525–546. 10.1113/jphysiol.1992.sp019243 1464843 PMC1175572

[B15] Fling BW, Walsh CM, Bangert AS, Reuter-Lorenz PA, Welsh RC, Seidler RD (2011) Differential callosal contributions to bimanual control in young and older adults. J Cogn Neurosci 23:2171–2185. 10.1162/jocn.2010.21600 20954936 PMC3809031

[B16] Goldberg G (1985) Supplementary motor area structure and function: review and hypotheses. Behav Brain Sci 8:567–588. 10.1017/S0140525X00045167

[B17] Grefkes C, Eickhoff SB, Nowak DA, Dafotakis M, Fink GR (2008) Dynamic intra- and interhemispheric interactions during unilateral and bilateral hand movements assessed with fMRI and DCM. Neuroimage 41:1382–1394. 10.1016/j.neuroimage.2008.03.048 18486490

[B18] Haller S, Chapuis D, Gassert R, Burdet E, Klarhöfer M (2009) Supplementary motor area and anterior intraparietal area integrate fine-graded timing and force control during precision grip. Eur J Neurosci 30:2401–2406. 10.1111/j.1460-9568.2009.07003.x 20092581

[B19] Hellebrandt FA (1951) Cross education; ipsilateral and contralateral effects of unimanual training. J Appl Physiol 4:136–144. 14888623 10.1152/jappl.1951.4.2.136

[B20] Hortobagyi T, Richardson SP, Lomarev M, Shamim EA, Meunier S, Russmann H, Dang N, Hallett M (2011) Interhemispheric plasticity in humans. Med Sci Sport Exerc 43:1188–1199. 10.1249/MSS.0b013e31820a94b8 PMC4137570 21200340

[B21] Houzel JC, Milleret C (1999) Visual inter-hemispheric processing: constraints and potentialities set by axonal morphology. J Physiol Paris 93:271–284. 10.1016/S0928-4257(00)80056-X 10574117

[B22] Kermadi I, Liu Y, Rouiller EM (2000) Do bimanual motor actions involve the dorsal premotor (PMd), cingulate (CMA) and posterior parietal (PPC) cortices? Comparison with primary and supplementary motor cortical areas. Somatosens Mot Res 17:255–271. 10.1080/08990220050117619 10994596

[B23] Kobayashi M, Hutchinson S, Schlaug G, Pascual-Leone A (2003) Ipsilateral motor cortex activation on functional magnetic resonance imaging during unilateral hand movements is related to interhemispheric interactions. Neuroimage 20:2259–2270. 10.1016/S1053-8119(03)00220-9 14683727

[B24] Koeneke S, Lutz K, Wüstenberg T, Jäncke L (2004) Bimanual versus unimanual coordination: what makes the difference? Neuroimage 22:1336–1350. 10.1016/j.neuroimage.2004.03.012 15219606

[B25] Kubota K, Hamada I (1978) Visual tracking and neuron activity in the post-arcuate area in monkeys. J Physiol Paris 74:297–312. 102777

[B26] Leemans A, Jones DK (2009) The B-matrix must be rotated when correcting for subject motion in DTI data. Magn Reson Med 61:1336–1349. 10.1002/mrm.21890 19319973

[B27] Leemans A, Jeurissen B, Sijbers J (2009) 17th Annual Meeting of International Society of Magnetic Resonance Med (Hawaii), ExploreDTI: a graphical toolbox for processing, analyzing and visualizing diffusion MR data, p3537. https://www.researchgate.net/profile/Alexander_Leemans/publication/288256284_ExploreDTI_a_graphical_toolbox_for_processing/links/5687c23908aebccc4e152812.pdf.

[B28] Montgomery LR, Herbert WJ, Buford JA (2013) Recruitment of ipsilateral and contralateral upper limb muscles following stimulation of the cortical motor areas in the monkey. Exp Brain Res 230:153–164. 10.1007/s00221-013-3639-5 23852324 PMC3778999

[B29] O'Connell RG, Balsters JH, Kilcullen SM, Campbell W, Bokde AW, Lai R, Upton N, Robertson IH (2012) A simultaneous ERP/fMRI investigation of the P300 aging effect. Neurobiol Aging 33:2448–2461. 10.1016/j.neurobiolaging.2011.12.021 22277263

[B30] Oldfield RC (1971) The assessment and analysis of handedness: the Edinburgh Inventory. Neuropsychologia 9:97–113. 10.1016/0028-3932(71)90067-4 5146491

[B31] Perez MA, Tanaka S, Wise SP, Sadato N, Tanabe HC, Willingham DT, Cohen LG (2007a) Neural substrates of intermanual transfer of a newly acquired motor skill. Curr Biol 17:1896–1902. 10.1016/j.cub.2007.09.058 17964167

[B32] Perez MA, Wise SP, Willingham DT, Cohen LG (2007b) Neurophysiological mechanisms involved in transfer of procedural knowledge. J Neurosci 27:1045–1053. 10.1523/JNEUROSCI.4128-06.2007 17267558 PMC6673204

[B33] Power JD, Barnes KA, Snyder AZ, Schlaggar BL, Petersen SE (2012) Spurious but systematic correlations in functional connectivity MRI networks arise from subject motion. Neuroimage 59:2142–2154. 10.1016/j.neuroimage.2011.10.018 22019881 PMC3254728

[B34] Raffelt D, Tournier JD, Rose S, Ridgway GR, Henderson R, Crozier S, Salvado O, Connelly A (2012) Apparent Fibre Density: a novel measure for the analysis of diffusion-weighted magnetic resonance images. Neuroimage 59:3976–3994. 10.1016/j.neuroimage.2011.10.045 22036682

[B35] Ruddy KL, Carson RG (2013) Neural pathways mediating cross education of motor function. Front Hum Neurosci 7:397. 10.3389/fnhum.2013.00397 23908616 PMC3725409

[B36] Ruddy KL, Leemans A, Carson RG (2016a) Transcallosal connectivity of the human cortical motor network. Brain Struct Funct. In press. 10.1007/s00429-016-1274-1 PMC5368198 27469272

[B37] Ruddy KL, Rudolf AK, Kalkman B, King M, Daffertshofer A, Carroll TJ, Carson RG (2016b) Neural adaptations associated with interlimb transfer in a ballistic wrist flexion task. Front Hum Neurosci 10:204. 27199722 10.3389/fnhum.2016.00204 PMC4853797

[B38] Tanji J, Shima K (1994) Role for supplementary motor area cells in planning several movements ahead. Nature 371:413–416. 10.1038/371413a0 8090219

[B39] Tax CM, Jeurissen B, Vos SB, Viergever MA, Leemans A (2014) Recursive calibration of the fiber response function for spherical deconvolution of diffusion MRI data. Neuroimage 86:67–80. 10.1016/j.neuroimage.2013.07.067 23927905

[B40] Uehara K, Morishita T, Kubota S, Funase K (2013) Neural mechanisms underlying the changes in ipsilateral primary motor cortex excitability during unilateral rhythmic muscle contraction. Behav Brain Res 240:33–45. 10.1016/j.bbr.2012.10.053 23174210

[B41] Veraart J, Sijbers J, Sunaert S, Leemans A, Jeurissen B (2013) Weighted linear least squares estimation of diffusion MRI parameters: strengths, limitations, and pitfalls. Neuroimage 81:335–346. 10.1016/j.neuroimage.2013.05.028 23684865

[B42] Werhahn KJ, Kunesch E, Noachtar S, Benecke R, Classen J (1999) Differential effects on motorcortical inhibition induced by blockade of GABA uptake in humans. J Physiol 517:591–597. 10.1111/j.1469-7793.1999.0591t.x 10332104 PMC2269337

[B43] Wilcox R (2012) Introduction to robust estimation and hypothesis testing, Ed 3 Amsterdam: Elsevier.

[B44] Winterer G, Adams CM, Jones DW, Knutson B (2002) Volition to action–an event-related fMRI study. Neuroimage 17:851–858. 10.1006/nimg.2002.1232 12377159

[B45] Zuo XN, Kelly C, Di Martino A, Mennes M, Margulies DS, Bangaru S, Grzadzinski R, Evans AC, Zang YF, Castellanos FX, Milham MP (2010) Growing together and growing apart: regional and sex differences in the lifespan developmental trajectories of functional homotopy. J Neurosci 30:15034–15043. 10.1523/JNEUROSCI.2612-10.2010 21068309 PMC2997358

